# Effects of serial NT-proBNP measurements in patients with acute decompensated heart failure: Results of the POC-HF pilot trial

**DOI:** 10.21542/gcsp.2024.31

**Published:** 2024-08-01

**Authors:** Maria Boesing, Frederick Bierreth, Kristin Abig, Stéphanie Giezendanner, Anne B. Leuppi-Taegtmeyer, Giorgia Lüthi-Corridori, Sabrina Maier, Stephanie Züsli, Jörg D. Leuppi, Thomas Dieterle

**Affiliations:** 1 University Institute of Internal Medicine, Cantonal Hospital Baselland, Liestal, Switzerland; 2 Faculty of Medicine, University of Basel, Basel, Switzerland; 3 Department of Patient Safety, Medical Directorate, University Hospital Basel, Basel, Switzerland

## Abstract

Introduction: Serial N-terminal pro-B-type natriuretic peptide (NT-proBNP) measurements have proven to be useful for therapy monitoring in patients hospitalized for acute decompensated heart failure (ADHF). The POC-HF pilot study investigated whether serial NT-proBNP measurements influenced treatment decisions in these patients.

Methods: Patients hospitalized for ADHF were randomly assigned to an intervention group (serial NT-proBNP measurements made available to treating physicians) or a control group (care as usual). HF therapy was administered at the discretion of the treating physician. The primary endpoint was dose changes in HF therapy during hospitalization. Secondary endpoints included changes in NT-proBNP levels, recovery from HF symptoms, length of hospital stay, and quality of life.

Results: 52 patients (35% female; mean age 81.8 years) were included. The availability of serial NT-proBNP values was associated with higher dosages of ACE inhibitors (relative treatment effect (RTE) day 11:0.74, *p* = 0.007) and loop diuretics (RTE day 11:0.77, *p* = 0.005), and lower dosages of beta-blockers (RTE day 11:0.43, *p*  =  0.002). NT-proBNP levels decreased (−752 pg/ml, *p* = 0.162) and recovery rates from ADHF symptoms were more pronounced in the intervention group, but without statistical significance. No differences were found in terms of the length of hospital stay and quality of life.

Conclusion: The results of this pilot trial indicate that serial NT-proBNP measurements are possibly associated with faster up-titration of HF medication, more pronounced NT-proBNP decrease, and faster recovery from symptoms than symptom-guided therapy in patients hospitalized for ADHF. These preliminary findings require further validation through larger studies.

Trial registration : http://www.swissethics.ch BASEC-ID 2017-01030, registered on 28 December 2017.

## Introduction

Heart failure (HF), a primary cause of morbidity and mortality in the Western world, represents a major social and economic burden on healthcare systems^1–3^. With its incidence progressively increasing with age above 50 years and a reported prevalence of over 8% in the age group 75+ years, it affects over 26 million patients worldwide^1,4,5^. Recurring acute decompensation results in repeated unplanned hospitalizations and is associated with poor prognosis^3,4,6^. A study assessing the global economic burden of HF with data from 197 countries estimated the overall cost of HF to be over $100 billion per year^6^.

N-terminal pro-B-type natriuretic peptide (NT-proBNP) has become a hallmark biomarker in patients with suspected acute decompensated HF (ADHF)^7–9^. In these patients, increased natriuretic peptide (NP) levels (BNP >100 pg/ml or NT-proBNP >300 pg/ml) can be used as a diagnostic tool in addition to typical symptoms (dyspnea, peripheral edema, jugular vein distention, and fatigue), signs of cardiomegaly and lung congestion on chest radiography, and echocardiographic changes such as left ventricular hypertrophy, dilation, and diastolic dysfunction^7,8^. Since increased NPs are not only diagnostic for HF but are also associated with increased mortality and morbidity, a decrease in these peptides may help to prevent readmissions and improve prognosis^10,11^.

According to the 2016 guidelines for the diagnosis and treatment of acute and chronic HF of the European Society of Cardiology (ESC), optimal pharmacological HF therapy consists of symptom management with diuretics, nitrates and/or digoxin, as well as the improvement of prognosis with angiotensin converting enzyme inhibitors (ACE I), angiotensin receptor blockers (ARB), mineralocorticoid receptor antagonists (MRA), and beta-blockers (BB)^8^. Because combined angiotensin receptor and neprilysin inhibition (ARNI) with sacubitril/valsartan was considered a prognostically beneficial therapy escalation in patients with HF with reduced ejection fraction (HFrEF)^8,12^, the 2021 ESC guidelines for the diagnosis and treatment of acute and chronic HF recommend ARNI as a replacement for ACE-I in these patients^13^. Several studies have demonstrated that the use of ACE-Is, BBs, ARBs, and MRAs in chronic HF is not only associated with an improved prognosis but also an overall decrease in serum NP levels^14–17^. Nevertheless, both in- and outpatients are often treated with dosages lower than recommended^18,19^.

The use of serial NT-proBNP measurements for disease and therapy monitoring of HF has been investigated in several clinical trials in the outpatient setting and in one trial in patients hospitalized for ADHF, leading to inconsistent conclusions^20–27^. Some studies have highlighted an association between natriuretic peptides and titration of ACE-I or MRAs in stable HF^25,26^. These observations give raise to the hypothesis that a similar association might be seen in the acute decompensated setting. The results of the recently published STRONG-HF trial demonstrated that rapid up-titration of guideline-directed HF medication and close follow-up after admission for ADHF symptoms improved quality of life and reduced the risk of 180-day all-cause death or HF readmission when compared with care as usual^28^. Rapid up-titration was also found to be safe and led to a more prominent decrease in NT-proBNP levels compared to care as usual^28^. Previous studies have demonstrated a beneficial prognostic effect of a decrease in serum NT-proBNP levels during hospitalization^10,11^. Despite the fact that pharmacological treatment may be influenced by serial NT-proBNP measurements, only sparse and conflicting data are available on its effects on dosage adjustment of guideline-directed HF medications in patients hospitalized for ADHF^8,18^. Therefore, this pilot study aimed to assess whether serial NT-proBNP measurements may influence treatment decisions, such as dose adjustments of prognostically beneficial HF medication, and outcomes in patients hospitalized for ADHF.

## Material and Methods

### Study design and objectives

Details of the rationale and design of the POC-HF study have been previously published^29^. This investigator-initiated, single-center, randomized controlled prospective pilot trial was conducted at the Cantonal Hospital Baselland, Liestal, between 2018 and 2020, and included 52 patients hospitalized for ADHF. The primary objective was to investigate the effect of the availability of serial NT-proBNP measurements on physicians’ treatment decisions. The secondary objective was to assess the effect of NT-proBNP monitoring on other prognostic parameters, such as the change in serum NT-proBNP level, recovery from signs and symptoms, quality of life (QoL), and length of hospital stay. The study was approved by the Ethics Committee of Northwestern and Central Switzerland (Ethikkommission Nordwest- und Zentralschweiz, EKNZ) and is registered at http://www.swissethics.ch (BASEC-ID 2017-01030) and http://www.clinicaltrials.gov (NCT04471610). The study was performed in accordance with the ethical standards of the 1964 Declaration of Helsinki and its later amendments. All the patients included in the study provided written informed consent.

**Table 1:** POC-HF eligibility criteria.

**Inclusion criteria**	**Exclusion criteria**
• NT-proBNP >300 pg/ml and creatinine clearance ≥ 60 ml/min or NT-proBNP >1200 pg/ml • NYHA functional class II or III • Symptoms of heart failure (at least one), i.e., - Dyspnea - Paroxysmal nocturnal dyspnea - Reduced exercise capacity - Fatigue - Extended recovery after exercising - Peripheral edema (lower leg, ankle) • Age >18 years • Written informed consent	• Requirement for immediate intensive care^a^• Uncontrolled brady- or tachyarrhythmia • Unstable angina pectoris • Severe uncorrected valvular disease • Planned cardiac intervention in the next 6 months • Clinically significant concomitant disease states • Active infection • Immunosuppressive medical therapy • Heart failure due to chemotherapeutic drugs • Known or suspected non-compliance, drug or alcohol abuse • Pregnancy or lactation • Participation in another interventional study • Investigators or their family members

**Notes.**

NT-proBNP N-terminal pro-B-type natriuretic peptide NYHA New York Heart Association

a This included life-threatening conditions that required immediate and close monitoring and advanced therapies only available in an intensive care setting.

### Study population

Eligibility criteria aimed at ensuring participant safety and balancing internal validity with real-world applicability. The study team aimed to ask all patients with ADHF who fulfilled the eligibility criteria outlined in Table 1 to participate within 24 h of admission. Patients with preserved (HFpEF), mid-range (HFmrEF), and reduced (HFrEF) ejection fraction (EF) were eligible for participation in the study. Patients with initial life-threatening conditions, that did not allow for the protocol inclusion, were excluded from the study. Due to internal staff shortages, patient recruitment had to be put on hold for 16 months between July 2018 and November 2019, which limited recruitment considerably. In addition, due to constraints regarding available research personnel, patient recruitment was only possible during the daytime from Monday to Friday.

### Outcomes

The primary longitudinal endpoint of the study was defined as a change in pharmacological HF therapy from patient inclusion to hospital discharge. This included type, dosing, and rapidity of therapy adjustments. Dosages for each HF medication group were expressed in dose equivalents of enalapril (ACE-I), candesartan (ARB), spironolactone (MRA), metoprolol (BB), metolazone (thiazide-like diuretics (TD)), and furosemide (loop diuretics (LD)). Dose equivalents were as suggested by the Federal Union of German Associations of Pharmacists (Bundesvereinigung Deutscher Apothekerverbände, ABDA)^30^. An average bioavailability of 50% was assumed for oral furosemide. Secondary endpoints were change of serum NT-proBNP, NYHA functional class, vital signs, length of hospital stay, weight loss, and QoL on two validated scales (Minnesota Living with Heart Failure Questionnaire (MLWHFQ), and 12-item Short Form Survey (SF-12)^31,32^). Safety outcomes included lab parameters such as serum potassium, sodium, and creatinine, as well as the occurrence of adverse events, such as transfer to intensive care unit and mortality. To minimize bias, all study-related measurements and procedures were performed in a centralized manner at the Cantonal Hospital Baselland in Liestal.

### Study procedures and randomization

All enrolled patients underwent an inclusion visit, during which general demographic and clinical data, including age, sex, nationality, smoking status, alcohol consumption, current profession, exercise behavior, allergies, medication, HF history, and comorbidities, were recorded. Additionally, vital signs, body weight, NYHA functional class, and QoL were assessed together with electrocardiogram and laboratory tests (NT-proBNP, potassium, sodium, and creatinine).

At inclusion, eligible patients were randomized in a 1:1 ratio to either the intervention group or the control group. Patients allocated to the intervention group underwent serial measurements of NT-proBNP, potassium, sodium, and creatinine every second business day until hospital discharge. The point-of-care Cobas 232 POC system^33^ and FUJI DRI-CHEM NX500 FV system were used for the analyses^34^. The results were sent to the treating physician on the same day. Additionally, the treating physician was alerted by phone if the NT-proBNP level did not decrease by at least 10% between two consecutive measurements. Physicians were instructed to treat patients according to the ESC guidelines; there was no explicit protocol for recommended actions based on the patients’ NT-proBNP results.

Patients allocated to the control group underwent care as usual according to ESC guidelines; thus, no serial NT-proBNP measurements were performed, and diagnostic and therapeutic decisions were mainly guided by clinical signs and symptoms. However, treating physicians could request additional NT-proBNP measurements if they found it necessary as part of usual care. In both groups, diagnostic and therapeutic decisions were left to the discretion of the responsible physician, based on the 2016 ESC guidelines for the diagnosis and treatment of acute and chronic HF, as established at the Cantonal Hospital Baselland, Liestal^8^. According to these guidelines, ARNI was not recommended as first-line therapy for HF at the time of recruitment. The study team provided no specific recommendations concerning therapy. All pharmacological therapy adjustments were documented during hospitalization. Laboratory parameters, vital signs, body weight, NYHA functional class, and QoL were assessed for a second time in both groups during the discharge visit, which took place one day before or on the day of hospital discharge.

### Sample size considerations

As this project was a pilot and feasibility study that faced the lack of availability of appropriate previous results, we abstained from formal sample size calculation^29^. The sample size of 52 was based on the number of patients hospitalized for ADHF in the Cantonal Hospital Baselland in previous years. Results from this study will allow the estimate of effect sizes for the determination of sample sizes for future trials.

### Statistical analysis

Results are presented descriptively as absolute and relative frequencies for binary variables, mean ± standard deviation (SD) for normally distributed variables, and median/interquartile range (IQR) for non-normally distributed variables. Intervention effects on continuous variables were assessed using the Student’s *t*-test if normally distributed or the Mann–Whitney U-test otherwise. Intervention effects on categorical variables were assessed using Pearson’s chi-square test with Yates continuity correction or Fisher’s exact test for small frequencies. Medication dosage equivalents for each medication group were analyzed using R with the package nparLD, which enables nonparametric longitudinal analyses^35,36^. The between-group variables (patient group with serial NT-proBNP measurements *vs.* care as usual) and the within-group variable (time with treatment days) were analyzed to determine the main group and time effects, as well as interaction effects. Relative treatment effects (RTE) were used as a measure of effect with the following interpretation: RTEs lie between 0 and 1. An RTE >0.5 indicates a tendency for participants in a subgroup to receive a higher dosage, whereas an RTE <0.5 indicates a tendency to receive a lower dosage than a randomly drawn subject and time from the whole data set. An RTE of 0.5 means no effect. Daily dosage equivalents were plotted against time, and the Wald test was used to measure the significance of the effects. Patients with adverse events were considered to be right-censored in the longitudinal analysis.

To detect and quantify intervention effects on laboratory values and QoL we performed an analysis of variance (ANOVA). If applicable, and the variables fulfilled the necessary assumptions, the analysis was adjusted for the respective baseline values in an analysis of covariance (ANCOVA)^37^. The use of ANCOVA with adjustment for the respective baseline values accounts for potential baseline imbalances between the two groups^37^. Assumptions were examined graphically (normally distributed residuals), by means of Levene’s test (homoscedasticity), and ANOVA (homogeneity of covariates and regression slopes for ANCOVA), respectively. Statistical analysis and graphical presentation were performed using R version 3.4.3^38^. Statistical significance level was at 0.05.

## Results

### Study population

**Figure 1.**
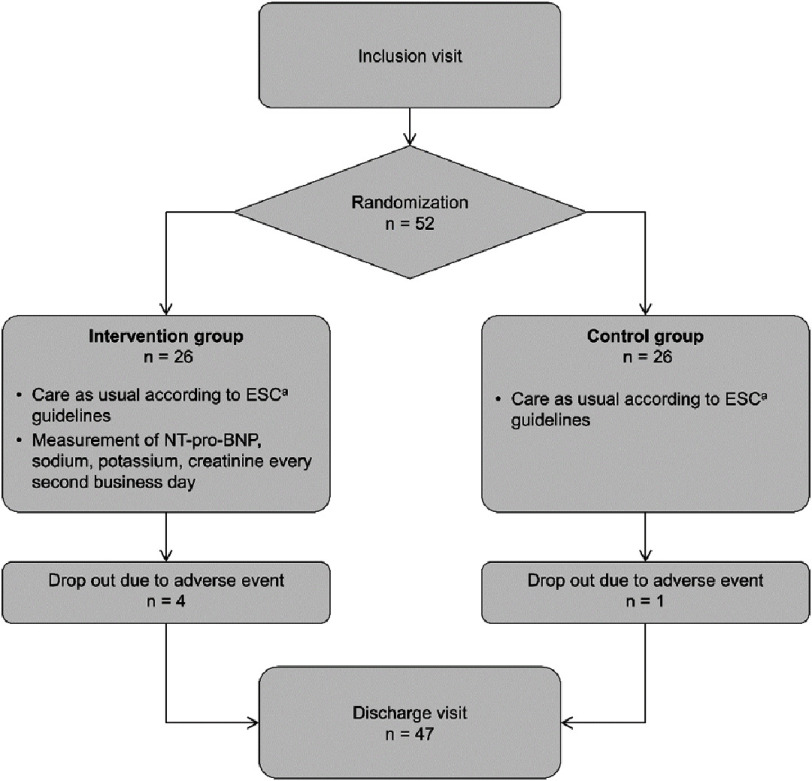
Number of patients per group from recruitment to discharge. ^a^ ESC: European Society of Cardiology.

Between January 2018 and May 2020, 223 patients with ADHF hospitalized at the Cantonal Hospital Baselland in Liestal, Switzerland were screened for eligibility, of which 52 were included in the study. A patient flow chart is provided in Figure 1.

Twenty-six patients were randomly allocated to each study arm. Forty-seven patients (90.4%) completed the study procedures per protocol, including the discharge visit, with a mean length of hospitalization of 7.9 days (SD ± 4.3). One patient (intervention group) had to be transferred to intensive care due to complications during a scheduled coronary angiography and subsequently died from respiratory deterioration. Four more patients (three from the intervention group and one from the control group) were transferred to intensive care due to acute bronchitis, acute state of confusion, renal impairment, and amiodarone intoxication, respectively. None of the adverse events were considered to be related to the study procedures. Patient characteristics at inclusion are shown in Table 2. No significant differences were observed between the intervention and control groups at the time of inclusion.

**Table 2:** Summary of patient characteristics at inclusion and discharge. Mean ±standard deviation for normally distributed variables, median (IQR) for non-normally distributed variables, and absolute frequencies (percentages) for binary variables.

	**Inclusion**					**Discharge**			
	**Intervention (*n* = 22)** ^**a**^	**Control** **(*n* = 25)** ^**a**^	***p*-value** ^**b**^		**Intervention (*n* = 22)** ^**a**^		**Control** **(*n* = 25)** ^**a**^	***p*-value** ^**b**^	
**Demographics**							
Female sex, *n* (%)	7 (31.8)	11 (44.0)	0.578^d^				
Age years	81.8 ± 7.3	79.2 ± 9.2	0.283				
Age years (range)	71–93	57–95					
**Clinical parameters**							
de-novo HF *n* (%)	7 (31.8)	8 (32.0)	0.764^d^				
HFrEF *n* (%)	5 (22.7)	6 (24.0)	0.808^d^				
HFmrEF *n* (%)	6 (27.3)	6 (24.0)	0.937^d^				
Weight kg	78.0 ± 15.8	81.0 ± 16.8	0.531		73.6 ± 15.3		76.0 ± 15.4	0.608	
Height cm	167.1 ± 9.5	169.1 ± 11.0	0.523				
BMI kg/m^2^	27.9 ± 5.0	28.3 ± 5.1	0.806		26.5 ± 4.9		26.7 ± 4.9	0.895	
NYHA class III, *n* (%)	13 (59.1)	18.0 (72.0)	0.533^d^		0 -		0 -	
**Vital signs**							
HR bpm	75.5 ± 14.1	83.9 ± 19.1	0.096		73.1 ± 13.6		76.2 ± 19.6	0.534	
SBP mmHg	134.2 ± 21.0	125.7 ± 19.7	0.159		125.7 ± 16.6		122.8 ± 17.9	0.577	
DBP mmHg	77 (67-86)	75 (64-81)	0.291^c^		75 (69-80)		67 (60-70)	0.026^c^*	
**Physical examination**							
Edema, *n* (%)	19 (90.5)	22 (88.0)	1.000^d^		7 (36.8)		12 (50.0)	0.580^d^	
Rales, *n* (%)	13 (61.9)	14 (56.0)	0.917^d^		2 (11.8)		7 (30.4)	0.256^e^	
Jugular vein distention, *n* (%)	11 (52.4)	15 (60.0)	0.825^d^		2 (11.1)		3 (14.3)	1.000^e^	
Hepatojugular reflux, *n* (%)	12 (57.1)	14 (60.9)	1.000^d^		4 (22.2)		4 (17.4)	0.713^e^	
SBP < 100 mmHg, *n* (%)	1 (5.0)	1 (4.0)	1.000^e^		2 (10.5)		3 (12.5)	1.000^e^	
3rd heart sound, *n* (%)	0 -	1(4.0)	1.000^e^		0 -		1 (4.4)	1.000^e^	
4th heart sound, *n* (%)	0 -	0 -			0 -		0 -	
**Lab parameters**							
NT-proBNP pg/ml	6820 (2773–11475)	3170 (2084–9001)	0.286^c^		3731 (1200–6747)		2085 (1464–6998)	0.716^c^	
Sodium mmol/l	141 (139–142)	141 (134–142)	0.739^c^		141 (139–143)		141 (137–142)	0.603^c^	
Potassium µmol/l	3.7 (3.5–4.2)	3.7 (3.4–4.1)	0.957^c^		3.8 (3.6–4.0)		3.8 (3.5-4.0)	0.604^c^	
Creatinine µmol/l	106 (89–144)	130 (97–164)	0.172^c^		106 (91-123)		116 (88–150)	0.692^c^	
eGFR ml/min/1.73 m^2^	39 (30–52)	44 (23–51)	0.609^c^		36 (30–52)		41 (28–52)	0.904^c^	
**Quality of Life**							
MLWHFQ score	46.7 ± 13.5	47.2 ± 13.7	0.905		33.7 ± 13.5		31.0 ± 15.2	0.555	
SF-12 Physical score	30.6 ± 8.8	29.9 ± 7.2	0.768		37.2 ± 9.1		38.4 ± 9.2	0.667	
SF-12 Mental score	54 (34–58)	53 (38–59)	0.782^c^		58 (52–62)		55 (51–58)	0.243^c^	

**Notes.**

* *p*-value < 0.05.

a patients treated per protocol.

b Intervention vs. control, student’s T-test unless otherwise stated (normally distributed).

c Intervention vs. control, Mann-Whitney-U-Test (not normally distributed).

d Intervention vs. control, Chi-SquaredTest with Yates continuity correction.

e Intervention vs. control, Fisher’s exact test (small sample).

HF Heart failure HFrEF Heart failure with reduced ejection fraction (<40%) HFmrEF Heart failure with mid-range ejection fraction (40-49%) BMI Body Mass Index NYHA New York Heart Association HR Heart rate SBP systolic blood pressure DBP diastolic blood pressure eGFR estimated glomerular filtration rate MLWHFQ Minnesota Living with Heart Failure Questionnaire SF-12 12-item Short Form Survey for health related quality of life

### Effects on HF medication

The summary of prescribed HF medication at study inclusion and discharge, including daily dosage equivalents (dd eq), are given in Table 3(a) and changes of dd eq during hospitalization in Table 3(b). HF medication at baseline included ACE-I, ARB, ARNI, MRA, BB, TD, and LD (Table 3(a)). At some point during hospitalization, all study participants were treated with LD, while TD were given additionally in 36% *vs.* 46% (*p* = 0.461) and MRA in 23% *vs.* 31% (*p* = 0.588) in intervention *vs.* control group. Absolute changes of dd during hospitalization were not significantly different in intervention and control group (Table 3(b)). While none of the patients received ARNI at hospital admission, this therapy was initiated during hospitalization in one patient in the control group. When comparing patients with HFrEF and HFmrEF (EF ≤ 50%) with patients with HFpEF (EF ≥ 50%), no significant differences in changes of dd eq were observed.

**Table 3:** Pharmacological heart failure therapy by medication group and study group. (A) Summary of prescribed HF medication at inclusion and discharge; (B) Changes of daily dosage equivalents from inclusion to discharge.

**(a)**					
	**Intervention (*n* = 22)** ^**a**^			**Control (*n* = 25)** ^**a**^	
	**Inclusion**	**Discharge**		**Inclusion**	**Discharge**
ACE-I, *n* (%)	11 (50%)	13 (59%)	** **	5 (20%)	6 (24%)
dd eq^c^ enalapril in mg	10 (10–15)	10 (10–10)	** **	5 (5-10)	6 (3-9)
ARB, *n* (%)	4 (18%)	4 (18%)		9 (36%)	6 (24%)
dd eq candesartan in mg	10 (4–18)	12 (7–16)		16 (8–16)	12 (8–16)
ARNI, *n* (%)	0	0		0	1 (4%)
dd eq entresto in mg					200.0
MRA, *n* (%)	6 (27%)	7 (32%)		5 (20%)	7 (28%)
dd eq spironolactone in mg	25 (25–38)	25 (13–38)		25 (25–25)	25 (25–25)
BB, *n* (%)	14 (64%)	13 (59%)		22 (88%)	20 (80%)
dd eq metoprolol in mg	88 (50–100)	75 (50–200)		100 (31–138)	100 (50–200)
TD, *n* (%)	3 (14%)	4 (18%)		7 (28%)	4 (16%)
dd eq metolazone in mg	0.6 (0.6–1.6)	2.5 (2.5–3.1)		2.5 (1.9–3.8)	1.9 (1.3–2.5)
LD, *n* (%)	19 (86%)	19 (86%)		24 (96%)	24 (96%)
dd eq furosemide in mg	53 (30–107)	80 (34–107)		63 (40–123)	53 (27–107)

**(b)**					
	**Intervention** ^**a**^	** **	**Control** ^**a**^	** **	***p*-value** ^**b**^
ACEI-I, Δ dd eq enalapril in mg	0 (0–5)		0 (0–2.5)		0.812
ARB, Δ dd eq candesartan in mg	0 (−2–1)		0 (−4–0)		0.428
ARNI, Δ dd eq entresto in mg	–		+200		–
MRA, Δ dd eq spironolactone in mg	+25 (25–44)		0 (−3–25)		0.745
BB, Δ dd eq metoprolol in mg	0 (0–0)		0 (0–0)		0.742
TD, Δ dd eq metolazone in mg	+1.3 (−0.5–2.5)		−0.6 (−3–0)		0.089
LD, Δ dd eq furosemide in mg	−13 (−40–46)		−7 (−13–0)		0.975

**Notes.**

Daily dosage equivalents are displayed as median (IQR); New installation/stop of a medication group during hospitalization was regarded as increase/decrease in daily dosage.

a patients treated per protocol.

b Intervention vs. control, Mann-Whitney U-Test (data not normally distributed).

dd eq daily dosage equivalent ACE-I angiotensin converting enzyme inhibitor ARB angiotensin II receptor blocker ARNI combined angiotensin receptor and neprilysin inhibitor MRA mineralocorticoid receptor antagonist BB Beta blocker TD Thiazide-like diuretic LD loop diuretic Δ change of daily dosage equivalent from inclusion to discharge

Table 4 presents the results of the longitudinal analysis for all HF therapy substance groups. Significant group-time interactions were found for the substance groups ACE-I (*p* = 0.007), BB (*p* = 0.002), and LD (*p* = 0.005), suggesting that dosing was affected by the intervention over time: ACE I and LD dosages were increased (RTEs day 11: ACE-I 0.74, LD 0.77), while BB dosages were decreased (RTE day 11: 0.43).

**Table 4:** Results of the longitudinal analysis: Group effect, time effect, and group-x-time interaction by medication group.

	**Group effect**			**Time effect**		**Group x Time interaction**	
ACE-I		0.008*			0.015^*^		0.007^*^
ARB		0.540			0.154		0.592
MRA		0.638			0.389		0.192
ARNI			*omitted due to lack of incidence*				
BB		0.198			0.023^*^		0.002^*^
TD		0.607			0.001^*^		0.111
LD		0.300			0.479		0.005^*^

**Notes.**

*P*-values corresponding to Wald-type statistics.

* *p*-value < 0.05.

ACE-I angiotensin converting enzyme inhibitor ARB angiotensin II receptor blocker ARNI combined angiotensin receptor and neprilysin inhibitor MRA mineralocorticoid receptor antagonist BB Beta blocker TD Thiazide-like diuretic LD loop diuretic

### Effects on serum NT-proBNP

Differences in secondary endpoints between the intervention and control group at discharge, adjusted for the respective baseline values, are presented in Table 5. In both groups, a decrease in serum NT-proBNP was observed, with a more pronounced decrease in the intervention group (−1851 *vs.* -903 pg/m, difference of mean adjusted for baseline NT-proBNP: −752 pg/ml, *p* = 0.162, see Table 5). Furthermore, in 40.9% of patients (9 of 22) in the intervention group and 36.0% (9 of 25) in the control group, a relative decrease in NT-proBNP level of 30% or more was observed during hospitalization (*p* = 0.73).

**Table 5:** Differences of mean change (before vs. after intervention) between groups with 95%- confidence intervals and p-value, adjusted for the respective baseline value if applicable.

	**Adjusted difference of mean change** ^a^	**95%-Confidence Interval**		**p-value**
		**Lower Limit**	**Upper Limit**	
**Clinical parameters**				
NT-proBNP pg/ml	−752	−1789	285	0.162
Weight kg	−0.5	−2.5	1.5	0.649
SBP mmHg	−1.3	−9.7	7.2	0.770
Length of hospital stay^b^ days	1.1	−1.3	3.5	0.361
**Safety parameters**				
Sodium mmol/l	−0.3	−2.6	1.9	0.780
Potassium *μ*mol/l	0.1	−0.2	0.3	0.646
eGFR ml/min/1.73m^2^	−2.6	−8.6	3.4	0.406
**Quality of Life**				
MLWHFQ score	2.4	−5.6	10.4	0.561
SF-12 Physical score	−1.3	−5.4	2.9	0.546
SF-12 Mental score	2.7	−1.5	6.8	0.218

**Notes.**

a Analysis of covariance (ANCOVA) was used to detect differences between the intervention and control group.

b Analysis of variance (ANOVA) was used to detect differences between the intervention and control group.

SBP systolic blood pressure eGFR estimated glomerular filtration rate MLWHFQ Minnesota Living with Heart Failure Questionnaire SF-12 12-item Short Form Survey

### Effects on length of hospital stay

The median length of hospital stay was 9 days (IQR: 5-11 days) in the intervention group and 7 days (IQR: 5–11 days) in the control group (Mann–Whitney U-test, *p* = 0.374). The mean difference, adjusted for age, sex, baseline NYHA functional class, and baseline serum NT-proBNP value was + 1.1 days in the intervention group (ANOVA *p* = 0.361, Table 5).

### Effect on clinical signs and symptoms

Recovery rates from typical signs and symptoms of ADHF by study group among patients who underwent physical examination at discharge were available in 43 patients. Four patients did not undergo physical examination according to the study protocol due to discharge during weekends or discharge on short notice. Most enrolled patients that underwent a discharge visit improved their NYHA functional class by at least one level throughout hospitalization (intervention group 85%, control group 92%, *p* = 0.488). The majority of patients recovered from the initial signs and symptoms of ADHF, and recovery rates were higher in the intervention group for all symptoms except hepatojugular reflux. In patients with NYHA functional class III at baseline, 42% of patients in the intervention and 33% in the control group improved functional status to NYHA functional class I (*p* = 0.643). The differences were not statistically significant though. A summary of recovery rates from signs and symptoms is presented in the appendix, Table 6.

When adjusted for the respective baseline value, overall weight loss and overall decrease of systolic blood pressure (SBP) during the hospitalization were marginally more pronounced in the intervention than in the control group (weight: −0.5 kg, *p* = 0.649, SBP: −1.3 mmHg, *p* = 0.770, see Table 5).

### Effects on quality of life

Adjusted for the respective baseline values, average SF-12 mental component scores improved slightly more in the intervention group, while SF-12 physical component scores and MLWHFQ scores improved more in the control group (Table 5). None of the differences was statistically significant.

### Safety and adverse events

While only marginal differences in changes of serum sodium and potassium were observed between the two groups, 69% of all patients experienced electrolyte imbalances, most frequently hypokalemia (44% in the intervention *vs.* 48% in the control group, *p* = 1.0). Larger changes were observed for glomerular filtration rate, with less favorable results in the intervention group (difference adjusted for baseline value: eGFR −2.6 ml/min/1.73 m^2^, *p* = 0.406, Table 5). However, these alterations were not statistically significant and did not result in severe adverse events.

## Discussion

Despite important advances in the pharmacological therapy of HF, therapy monitoring is still based on signs and symptoms of this syndrome. Several trials have investigated the value of serial measurement of natriuretic peptides for disease and therapy monitoring, as well as therapy guidance in HF in the outpatient setting, demonstrating mixed results^20,22–26,39^. In particular, the largest trial investigating the effects of HF therapy guidance with repetitive NT-proBNP measurements, the GUIDE-IT trial, failed to demonstrate superiority of this approach over care as usual^40^. Nevertheless, NT-proBNP was repeatedly demonstrated to be a strong and independent predictor of morbidity and mortality in patients with HF and a decrease in NT-proBNP during hospitalization for ADHF represents a beneficial prognostic factor^10,11^. However, so far no data are available on the effects of repetitive NT-proBNP measurements in patients hospitalized for ADHF^8,18^.

We therefore conducted the POC-HF pilot study with the goal to assess whether serial NT-proBNP measurements influence treatment decisions, such as dose adjustment of prognostically beneficial HF medication, and outcomes in patients hospitalized for ADHF. While additional NT-proBNP measurements were allowed and left at the discretion of the treating physicians in the control group, serial measurements were only performed in the intervention group. A post-hoc analysis demonstrated that on average less than one additional NT-proBNP measurement was performed per patient after study inclusion in the control group (0.4 measurements), compared to 3.7 measurements in the intervention group. Therefore, additional NT-proBNP measurements in the control group are unlikely to have introduced a bias into the study.

Testing for group and time effects, as well as group-time interactions revealed that up-titration of pharmacological treatment with ACE-I and LD was more likely, while up-titration of BB was less likely in the intervention group compared to the control group. Average NT-proBNP at baseline was numerically, though not statistically significantly, higher in the intervention group than in the control group. The overall decrease in NT-proBNP was more pronounced in the intervention group than in the control group. Similar effects were observed for the recovery from signs and symptoms of ADHF. No signs of an increased risk for serious adverse events with the availability of NT-proBNP were observed when compared with symptom-guided HF therapy. However, the availability of serial NT-proBNP measurements did not result in a shortened duration of hospitalization when compared to care as usual.

Compared to the PRIMA II study, the only study so far investigating NT-proBNP-guided therapy *vs.* care as usual in patients hospitalized for ADHF, a different approach was used in POC-HF: First, patients were included into the study immediately after hospitalization and without prior stabilization^27,29^. Second, per protocol there was no requirement to achieve a specific reduction of serum NT-proBNP. However, treating physicians were specifically informed when an NT-proBNP reduction of <10% was observed between two consecutive measurements^27,29^. Third, decisions on HF diagnostic and therapeutic procedures, as well as the decisions on timing for hospital discharge and follow-up recommendations were left at the discretion of the responsible physician. Thus, no predefined treatment algorithm had to be followed^27,29^. For this reason, neither sample size nor power calculations were possible at the time of the POC-HF study set up.

Patients in POC-HF were slightly older than in PRIMA II^27^. While baseline NT-proBNP values in the intervention group of POC-HF were comparable to the baseline values in PRIMA II, the average NT-proBNP was numerically, though not statistically significantly lower in the control group^27^. This may indicate a selection bias and at least partially explain the higher likelihood for increasing the dosages of ACE-I and LD and the higher dose of TD in the intervention group, which in turn might explain the more pronounced reduction of serum NT-proBNP in the intervention group. However, unlike PRIMA II, this pilot trial did not include an initial run-in phase with the goal of clinical stabilization. Such a run-in phase would certainly have led to an overall reduction of NT-proBNP values, thereby reducing variability, and potentially bringing initially differing values into line.

According to the ESC guidelines, prognostically relevant HF therapies should be up-titrated to the maximum tolerated dose effect in HF patients with HFrEF in order to achieve their best effect^8,13^. In fact, a more rapid and more pronounced up-titration of ACE-I was observed in POC-HF with available NT-proBNP measurements. Though the average dose of BB was increased, this drug class was less likely to be up-titrated with available serial NT-proBNP measurements. This observation might be related to the notion that increased NT-proBNP values are due to increased ventricular wall tension, while ACE-I and LD, in particular together with TD, may decrease wall tension more than BB. It has to be kept in mind that no prognostically relevant HF therapies existed for patients HFpEF at the time of inclusion in the POC-HF pilot study. Therefore, it may very well be that focusing on patients with HFrEF or HFmrEF alone would have led to a more pronounced effect with regards to up-titration of renin-angiotensin-aldosterone system (RAAS) inhibitors and BB. However, we did not detect significant differences between HFrEF/HFmrEF and HFpEF patients with regards to up- respectively down-titration of RAAS inhibitors or BB.

A decrease in NT-proBNP of 30% or more during hospitalization is associated with improved HF outcomes such as lower rehospitalization rates, morbidity, and mortality^10,11^. Even though the difference in our study was not statistically significant, an NT-proBNP decrease of ≥ 30% was met by 40.9% of patients in the intervention group *vs.* 36.0% in the control group, confirming results from previous studies demonstrating that NT-proBNP monitoring leads to a prognostically relevant serum NT-proBNP decrease^25,26,39,41^.

As in PRIMA II, we did not observe differences between the two groups in terms of length of hospital stay and improvement of short-term QoL^27^. However, recovery from signs and symptoms of ADHF, such as edema, rales, and jugular vein distension tended to be more common in the intervention group compared to the control group.

Even though safety was not compromised and all AEs were considered unrelated to the study procedures, we observed a slight decrease in kidney function in the intervention group, while no changes were observed in the control group. Taking into account the results of the TIME-CHF study that pointed to a potential harmful effect of BNP-guided intensified HF therapy in patients older than 75 years^24^, the results of POC-HF underline the importance of close surveillance of safety parameters and individualized therapy protocols.

In PRIMA II, NT-proBNP guided HF therapy did not improve 6-month mortality or readmissions rates^27^. Due to the fact that POC-HF was a pilot study, no conclusions on longer-term effects of serial NT-proBNP measurements on rehospitalization, morbidity or mortality can be drawn. Further studies with larger sample sizes are needed to assess the respective effects.

Several limitations need to be mentioned. Inclusion was based on elevated levels of NT-proBNP together with signs and symptoms typical for heart failure. Thus, patients with HFrEF, HFmrEF, and HFpEF were included in the study, inducing heterogeneity with regards to the study population. This introduced variability with regards to diagnostic and therapeutic strategies selected during hospitalization, possibly obscuring the effects of the intervention. While we think that the overall results reflect the current situation of heart failure care, in particular in regional or local hospitals, the heterogeneity of the study population regarding EF would technically require sub-group analyses, which are, however, unsubstantial due to the small sample size. To avoid the described bias, future studies should consider focusing on a more homogeneous population (*e.g.*, only HFrEF).

With POC-HF being a pilot study, the small sample size of 52 patients did also not result in sufficient power to detect in-between group differences for all major endpoints. Due to the nature of the intervention, it was not possible to blind treating physicians. Patients were not actively told their group allocation, but due to blood sampling patterns in the intervention group, patients might have guessed their respective group allocation. The necessity of excluding five individual patients from the analysis due to transfer to different organizational units may have introduced a selection bias. Furthermore, a follow-up to assess patients’ mid-term outcomes including rehospitalizations, mortality, cardiovascular events and QoL was not performed, which limits the clinical implications of the results. Including mid- to long-term clinical endpoints in future study designs, such as 30-day or 3-months rehospitalization and mortality, could provide valuable information.

## Conclusion

In conclusion, the results of the POC-HF pilot study indicate that serial NT-proBNP measurements are associated with faster up-titration of HF medication, more pronounced decrease in NT-proBNP, and faster recovery from signs and symptoms when compared to symptom-guided therapy in a real-world patient population hospitalized for ADHF. The relatively small number of patients included into the POC-HF pilot study limit the generalizability of the results to the entire ADHF population. The results are therefore preliminary and require validation in larger studies. Trials including long-term follow-up and clinical endpoints such as rehospitalization and mortality are certainly needed to draw conclusions for clinical practice. Moreover, interventions based on standardized therapy protocols according to individual NT-proBNP target levels need to be investigated for further optimization of HF therapy.

## Statements and Declarations

**Ethics approval:** The study was conducted in accordance with the Declaration of Helsinki, and approved by the Ethics Committee of Northwestern and Central Switzerland (Ethikkommission Nordwest- und Zentralschweiz, EKNZ) and is registered at http://www.swissethics.ch (BASEC-ID 2017-01030) and http://www.clinicaltrials.gov (NCT04471610). All the patients included in the study provided written informed consent.

## Competing Interests

TD received a project grant from SynlabSuisse AG (Switzerland). JDL reports grants from Swiss National Science Foundation (SNF 160072 and 18559), as well as unrestricted grants from Astra Zeneca AG Switzerland, GSK AG Switzerland, and Sanofi AG Switzerland. Both TD and JDL received a project grant within the Swiss Personalized Health network Driver Project CREATE-PRIMA (Project No. 2018DRI08). The authors declare that the research was conducted in the absence of any commercial or financial relationships that could be construed as a potential conflict of interest.

## Data availability

The datasets generated during and/or analyzed during the current study are not publicly available due to data protection reasons but are available from the corresponding author on reasonable request.

## Author contributions

**Conceptualization & Design**: Frederick Bierreth, Stephanie Züsli, Jörg D. Leuppi, Thomas Dieterle

**Data Acquisition**: Maria Boesing, Kristin Abig, Frederick Bierreth, Sabrina Maier, Stephanie Züsli

**Data Analysis & Interpretation**: Maria Boesing, Thomas Dieterle, Stéphanie Giezendanner

**Writing - Original Draft Preparation**: Maria Boesing, Thomas Dieterle

**Writing - Review & Editing**: Kristin Abig, Frederick Bierreth, Stéphanie Giezendanner, Anne B. Leuppi-Taegtmeyer, Giorgia Lüthi-Corridori, Sabrina Maier, Jörg D. Leuppi, Thomas Dieterle

**Administrative, Technical & Laboratory Support**: Kristin Abig, Sabrina Maier

**Study Supervision**: Thomas Dieterle, Jörg D. Leuppi.

## Acknowledgements

We are grateful for the generous technical support provided by Stefan Kaspar from One provide AG, Frauenfeld, Switzerland. We are also grateful to Stefan Hubeli, Alexander Souza, and Hansjörg Lehner, for providing expert IT support concerning the implementation of laboratory devices into the existing hospital IT network as well as for providing software solutions applied in this study. Furthermore, we thank Zahra Pasha for her valuable language editing.

## Funding sources

Start-up funding for this study was provided by a project grant from Synlab Suisse AG, Lucerne, Switzerland. Further funding was provided within the Swiss Personalized Health Network Driver Project CREATE PRIMA (Clinical Research from multi-modality big data sources without proprietary interfaces in a multicenter approach, Project No. 2018DRI08). Laboratory devices including consumables were provided by One provide AG, Frauenfeld, Switzerland, an affiliated company of Synlab. IT support was provided by Iterata Medical Systems AG, Gränichen, Switzerland.

## Appendix

**Table 6:** Recovery from symptoms of acute decompensated heart failure by study group.

	** **	**Present at Inclusion** ^a^			**Recovered at Discharge**				
** **	** **	**Intervention (*n* = 19)**	**Control (*n* = 24)**	** **	**Intervention (*n* = 19)**		**Control (*n* = 24)**		**p-value** ^b^
Severe dyspnea^c^		12	18		5	(42%)	6	(33%)	0.643
Edema		17	21		11	(65%)	10	(48%)	0.292
Rales		10	13		8	(80%)	7	(54%)	0.192
Jugular vein distention		10	13		8	(80%)	10	(77%)	0.859
Hepatojugular reflux		11	14		7	(64%)	10	(71%)	0.678
3rd heart sound		–	1		–		0	(0%)	–

**Notes.**

a Patients, who underwent physical discharge examination.

b Recovered at Discharge Intervention vs. Control, Chi-Squared test with Yates continuity correction.

c NYHA III.

SBP systolic blood pressure eGFR estimated glomerular filtration rate MLWHFQ Minnesota Living with Heart Failure Questionnaire SF-12 12-item Short Form Survey
